# The Effects of Excessive Arousing Video Gaming on vmHRV During Sleep in Habitual Gamers

**DOI:** 10.1007/s10484-025-09723-z

**Published:** 2025-07-05

**Authors:** André Alesi, Kristina Klier, Benedict Herhaus, Klara Brixius, Ingo Froböse, Matthias Wagner, Katja Petrowski

**Affiliations:** 1 Institute of Movement Therapy and Movement-Oriented Prevention and Rehabilitation, German Sport University, Am Sportpark Müngersdorf 6, 50933 Cologne, Germany; 2 Institut Für Schlaf Und Regeneration GmbH, Schramberg, Germany; 3 Faculty of Computer Science, Institute of Computer Science, University of the Bundeswehr Munich, Munich, Germany; 4 Medical Psychology & Medical Sociology, University Medical Center Mainz, Johannes Gutenberg University of Mainz, Mainz, Germany; 5 Institute of Cardiology and Sports Medicine, Germany Sports University, Cologne, Germany; 6 Faculty of Human Sciences, Institute of Sports Science, University of the Bundeswehr Munich, Munich, Germany

**Keywords:** Heart rate variability (HRV), Vagally-mediated HRV (vmHRV), Competitive gaming, Computer game, Sleep

## Abstract

This study examines the impact of highly arousing video gaming compared to passive digital media consumption on vagally-mediated heart rate variability (vmHRV) and heart rate (HR) during subsequent sleep in healthy young men. Using a randomized, counterbalanced within-subject design, 31 habitual gamers (mean age: 23 years; BMI: 25.68; gaming experience: 8.69 years; daily gaming time: 1.96 h) alternated between two conditions: 120 min of evening video gaming or watching a nature film of equivalent duration, on two consecutive days per condition. Electrocardiogram (ECG) data collected during sleep revealed that while both conditions were associated with an increase in vmHRV, indicative of parasympathetic recovery, this increase was significantly greater following the film condition (RMSSD: t(446.1) = 2.05, *p* = 0.04; HF-HRV: t(446.1) = 2.00, *p* = 0.05). The marginal R^2^ values (RMSSD: 0.051, HF-HRV: 0.042) indicate that while the effect is present, it is moderate. No significant differences in HR were detected between conditions, supporting the idea that vmHRV is a more sensitive marker of autonomic modulation than HR. These findings underscore the potential of evening digital activity to modulate autonomic recovery processes. Specifically, highly stimulating gaming may impede parasympathetic activation, compared to the restorative effects of passive media consumption. This study contributes to the understanding of physiological responses to digital engagement and highlights the importance of mindful pre-sleep activities.

**Supplementary Information:**

The online version contains supplementary material available at 10.1007/s10484-025-09723-z.

## Introduction

 The increasing importance of video games (VGs) in modern culture has become evident over the past 30 years. This transformation has been extensively documented, highlighting video games as an omnipresent component of today's society (Toth et al., 2020 ). The results of the Digital-Media-Trends survey 2022 indicate that over 80% of U.S. respondents engage in video gaming, with half of smartphone owners playing on their mobile devices every day (Westcott et al., 2022 ). According to Statista 2023, the video game sector generated revenues of $319.91 billion in 2022 and is expected to experience an annual growth rate of 7.80%, projecting a market volume of $502.40 billion by 2027 (Statista, 2023 ). Video Gaming is predominantly an evening or nighttime activity, largely because daytime commitments such as work, school, and other responsibilities leave limited opportunities for recreational activities during the day (Kemp et al., 2021 ).

VGs are an integral part of daily life and have varied effects on physical health, particularly regarding sleep and stress regulation. Their impact on sleep quality and stress levels is complex, with stress often measured through changes in heart rate (Higuchi et al., 2005; Weaver et al., 2010). While the overall influence of VGs on sleep remains inconclusive, most studies report only minor effects (De Rosa et al., 2024; Klier et al., 2024). Specifically, playing stimulating VGs before bedtime may delay sleep onset and reduce sleep efficiency. In contrast, moderate VG use, defined as up to 7 h per week with low-intensity games, is not linked to adverse sleep outcomes (De Rosa et al., 2024).

Sleep is a complex state that involves significant changes in the hormonal and physiological regulation of heart activity (Zielinski et al., 2016). Within this regulatory framework, heart rate variability (HRV) emerges as a pivotal biomarker (Stein & Pu, 2012). HRV, defined by the temporal fluctuations between successive heartbeats, provides a quantifiable measure of autonomic nervous system function (Quigley et al., 2024). Heart rate (HR), on the other hand, reflects the combined influence of both the sympathetic nervous system (SNS) and parasympathetic nervous system (PNS), offering broader insights into autonomic balance. The simultaneous measurement of HR and HRV provides complementary information, allowing for a nuanced understanding of autonomic regulation (Quigley et al., 2024). While most HRV parameters reflect a mixed contribution from both the sympathetic nervous system (SNS) and parasympathetic nervous system (PNS), specific HRV parameters, such as vagally-mediated HRV (vmHRV), uniquely reflect the vagal contribution to cardiac function (Laborde et al., 2017). In addition, the high-frequency band (HF) of the HRV power spectrum which coincides with respiration (typically 0.15–0.4 Hz, called the respiratory band) and the root mean of the square successive differences (RMSSD) are of interest (Laborde et al., 2017).

The Vagal Tank Theory by Laborde et al. (2018) builds on this understanding by framing vmHRV as an indicator of self-regulatory efficiency. The model introduces three systematic levels of cardiac vagal control—resting, reactivity, and recovery—conceptualized through the metaphor of a "vagal tank" that can be depleted or replenished based on the body's demands (Laborde et al., 2018). HRV, particularly vmHRV, serves as a measure of the body's adaptive capacity. While higher vmHRV is associated with better emotional regulation, cognitive performance, and health outcomes (Thayer et al. 2009), reactivity and recovery provide insights into how effectively the body responds to and recovers from stressors (Stanley et al., 2013). HRV serves as a non-invasive indicator of autonomic regulation during different sleep stages and provides important insights into the connection between brain and cardiac activity (Chouchou & Desseilles, 2014). During non-rapid eye movement sleep (non-REMS), parasympathetic activity predominates, accompanied by reduced sympathetic modulation, which correlates with decreased brain activity in subcortical and cortical regions (Desseilles et al., 2008; Thayer et al., 2012). In contrast, rapid eye movement sleep (REMS) exhibits sympathetic predominance, associated with increased activity in central brain structures such as the amygdala and insular cortex (Critchley & Harrison, 2013; Desseilles et al., 2006).

Methodological inconsistencies and a lack of theoretical grounding hinder the comparability of studies on HRV in both traditional sports and esports (Welsh et al., 2023). Variations in measurement techniques, differences in participant populations, and inconsistent application of theoretical models often result in conflicting findings (Mosley & Laborde, 2022). A systematic review (Welsh et al., 2023) highlights that HRV studies in esports provide preliminary evidence of autonomic regulation during and after competitive gaming. For example, professional players exhibit higher HRV parameters, such as RMSSD or HF-HRV, after competition compared to amateur players, which may suggest more efficient recovery or adaptive regulation. However, methodological inconsistencies, such as variations in timing and measurement devices, limit the comparability and generalizability of these findings.

Ivarsson et al. (2009) showed that violent gaming increased HRV components both during gameplay and the subsequent night, without notable effects on subjective sleep quality (Ivarsson et al., 2009). A later study in 2013 revealed that less experienced gamers exhibited higher heart rate and LF/HF ratio during sleep after violent gaming, along with reduced subjective sleep quality and heightened negative emotions (Ivarsson et al., 2013). Furthermore, systematic review by De Rosa et al. (2024) highlighted that the effects of video gaming on sleep are modulated by the level of arousal induced by the game, session duration, and gaming frequency. Habitual and casual gaming were generally not associated with poor sleep, while excessive or highly arousing gaming, especially before bedtime, was linked to delayed sleep onset, reduced sleep efficiency, and poorer sleep quality (De Rosa et al., 2024). A recent study extended this research by focusing on habitual adult gamers (Long et al., 2023). They demonstrated that gaming led to a reduction in HF-HRV and RMSSD during gameplay, indicating attenuated parasympathetic activity. While the LF/HF ratio was also elevated, its interpretation as a direct marker of sympathovagal balance remains controversial, as it is influenced by multiple factors, including baroreflex function as well as both sympathetic and parasympathetic activity (Billman, 2013).

Building on these findings, the current study seeks to address critical gaps in understanding the physiological effects of gaming versus passive digital consumption on sleep. While prior research has predominantly focused on adolescents or failed to use consistent and validated HRV parameters, this study aims to investigate how the gaming condition and the film condition influence vagally mediated HRV (vmHRV) and heart rate (HR) during sleep in healthy young adults. By focusing on recognized HRV measures like RMSSD and HF-HRV (Laborde et al., 2017), the study aims to provide robust and standardized insights into autonomic regulation after digital engagement. This study hypothesizes that gaming before bedtime will decrease vmHRV and increase HR during sleep, reflecting sympathetic activation, whereas watching a nature film will enhance vmHRV and lower HR, promoting parasympathetic recovery.

## Materials and Methods

### Study Participants

Thirty-three male players aged 18–37 were initially recruited for the study. However, two datasets were excluded due to unusable ECG recordings, resulting in a final sample size of 31 participants (age: *M* = 23.00 ± 3.53years, BMI: *M* = 25.68 ± 3.34, time of experience: *M* = 8.69 ± 4.74 years, and daily gaming time: *M* = 1.96 ± 1.32 h). Only male participants were included in this study to reduce physiological variability in HRV and sleep parameters. Previous studies have shown sex-related differences in autonomic modulation, with women under 40 exhibiting lower sympathetic activity compared to men (Ramaekers, 1998). Furthermore, a recent systematic review and meta-analysis reported significant within-person fluctuations in cardiac vagal activity (a key component of HRV) across different phases of the menstrual cycle, highlighting the need to control for cycle phase in HRV-related research (Schmalenberger et al., 2020). While this exclusion was necessary to reduce physiological variability and strengthen internal validity, it should be noted that understanding female sleep physiology remains an important goal for future research.

The study participants were recruited through online tendering and notice boards at the authors institution. After recruitment, study inclusion and exclusion criteria were checked in a standardized interview. Inclusion criteria were being a male between 18 and 65 years, playing the games *League of Legends* (Riot Games, 2009) or *Counter Strike: Global Offensive* (Valve, 2012) and not being a professional e-sport player, in the definition not to earn personal salary by playing videogames. Exclusion criteria were any acute and/or chronic medical illness, mental disorders, or the use of medications that affect heart rate or the central nervous system, such as beta blockers. Additionally, participants who used perception-altering substances (e.g., certain pain relievers, recreational drugs) or medications that specifically alter cardiovascular function were excluded from the study. Rationale for Game Selection: *League of Legends* and *Counter Strike: Global Offensive* were chosen due to their popularity and their ability to generate high levels of cognitive engagement and competitive pressure, essential for inducing stress-related changes in HRV. Both games require complex decision-making and teamwork, providing ideal conditions to measure physiological stress responses. Other game genres that do not require real-time decision-making or intense competitive engagement were excluded to maintain the study's focus on high-stress digital environments. The study was conducted according to the guidelines of the Declaration of Helsinki and approved by the Ethics Committee of the University of the Bundeswehr Munich, Germany (05/28/2022). All participants were informed of the inherent risks and benefits of the study before signing an informed consent form.

### Experimental Design

The study was designed as a randomized within subject design. The entire study was conducted over a 6-month period to allow for the recruitment and testing of all participants. However, each participant was individually assessed within a tightly controlled 2-week window. During these 2 weeks, participants underwent the planned interventions and measurements. Therefore, interim health checks or long-term monitoring over the entire study duration were not necessary, as the health data of participants were collected solely during their specific assessment period. Among all participants, a 100€ and a 50€ voucher were raffled.

**Fig. 1:**
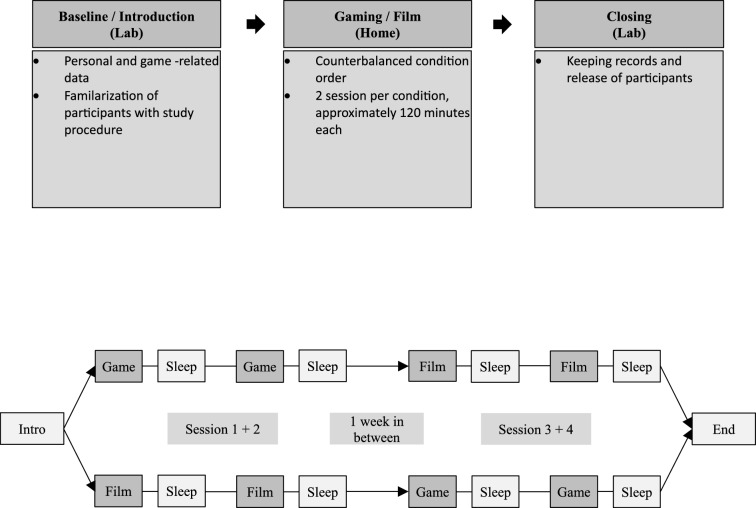
Flow chart of the examined study protocol

**Fig. 2:**
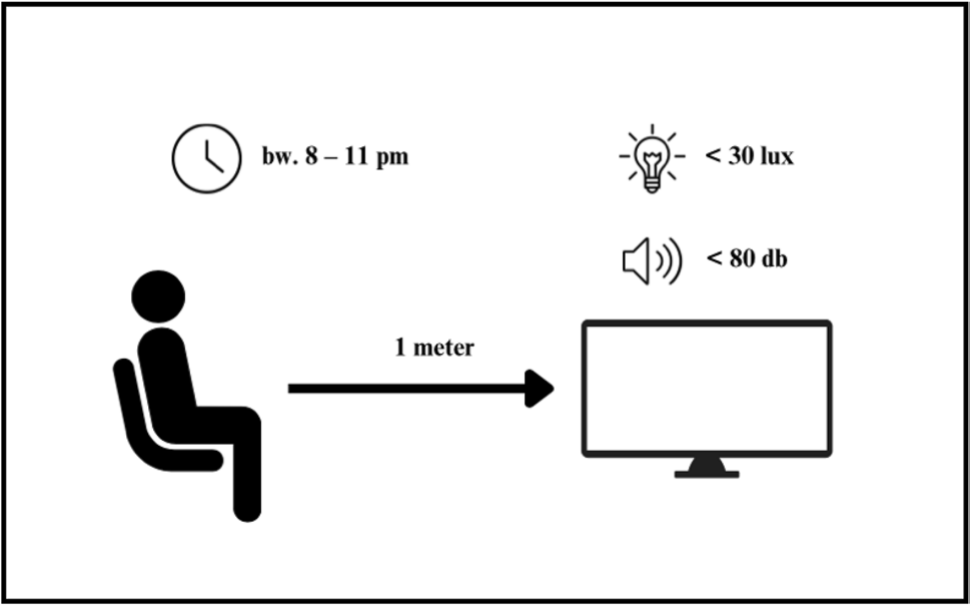
Experimental setup during the gaming and film conditions, showing the participant's distance from the screen (1 m), controlled lighting conditions (< 30 lux), sound level (< 80 dB), and time window (between 8 and 11 PM)

After the participants had agreed to the study, formalities had been clarified, explaining how to fil the daily log and answer all open questions. Then the participants were randomly divided into two groups. Group A starts in week 1 with two consecutive days of 120 min gaming and in week 2 with two consecutive days of 120 min watching a film. Group B started in the opposite order. The time difference between condition 1 and condition 2 was exactly one week. To compare the HRV measurements between the gaming and the film condition, and to ensure that the same activities are carried out during the day, participants were asked to wear the ECG-Sensor for 48 h each condition (see Fig. 1). The investigations were conducted between 8:00 PM and 11:00 PM. For the manipulation check, a logbook was provided to each participant, in which the exact start and end times of the intervention within the designated time window had to be documented. Participants were strictly instructed to refrain from consuming alcohol, caffeine, energy drinks, nicotine, and stimulating teas for 24 h prior to the experiment, to minimize any short-term effects on heart rate and heart rate variability. During the experiment, participants were allowed to drink, but only beverages that did not contain these substances. The participants could freely choose for gaming condition between two videogames (*Counter Strike: Global Offensive*; Valve, 2012 or *League of Legends*; Riot Games, 2009) for the study. The DVD *Earth* (Universal Film, 2007) and *Earth: One Amazing Day* (Universal Film, 2017) was used for the control condition. Participants were instructed to have their last meal until 7pm an check their environment parameters 30min before starting. Given that both light stimulation and posture can influence alertness in both conditions (Zeitzer et al., 2000), dim light conditions were held constant < 30 lux, distance from the television monitor (an additional light source) was controlled (1 m), subject were instructed to maintain a semi supine posture, and the volume was controlled to a speaker volume (< 80 db). For both conditions a normal pc or laptop were used (see Fig. 2).

### HRV Recording and Analysis

HRV was recorded using a portable single-channel electrocardiograph (ECG) device (Bittium Corporation, Faros™180, Oulu, Finnland). This sensor, a small portable rechargeable unit, was applied on the chest where it registered (and stored) the interbeat intervals measured from the R-wave maximum of a beat to the R-wave maximum of the next, with a sampling frequency of 1,000 Hz. HRV indices were analyzed using the Kubios™ HRV software (Biomedical Signal Analysis Group, Department of Applied Physics, University of Kuopio, Kuopio, Finland). All recorded ECGs (31 participants × 4 measurements = 124) were checked by experienced personnel beat-to-beat. Artefacts were corrected using the deletion method. The rate of losses (number of heartbeats marked as artifact or ectopic beats divided by all heartbeats detected, in %) was < 0.06%. The dependent variables were analyzed in terms of frequency (low frequency [LF], high frequency [HF]) and time (beat-to-beat intervals [R-R], standard deviation of the mean of the qualified NN interval [SDNN]. The proportion of consecutive NN intervals with a difference greater than 50 ms [pNN50] and the root mean square difference of consecutive normal RR intervals [RMSSD] were also measured (Johnston et al., 2020).

### Data Analysis

The sample size was determined through power calculations conducted with G*Power v.3.1 (Faul et al., 2009). Based on a medium effect size of Cohen´s *f* = 0.25, two conditions (film vs. gaming), *n* = 4 repetitions, a significant level of *p* = 0.05 and power of 80% (1− ß = 0.80), a total sample size of *N* = 24 was required for a two-way repeated measures ANOVA (time x group interaction effect).

**Fig. 3:**
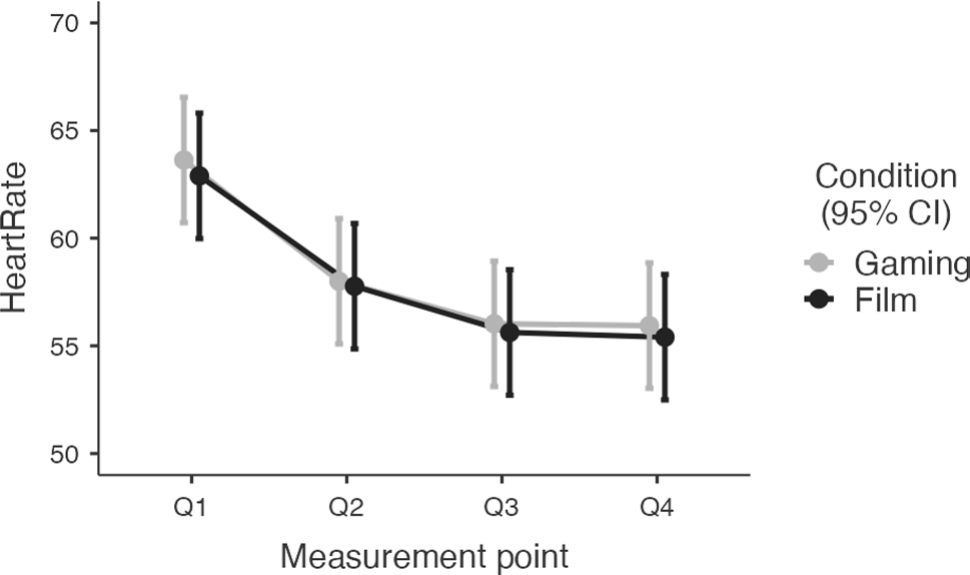
Heart Rate responses to four measurement points. Q1 was quarter one, Q2 was quarter two, Q3 was quarter three, Q4 was quarter four of the sleep time

**Fig. 4:**
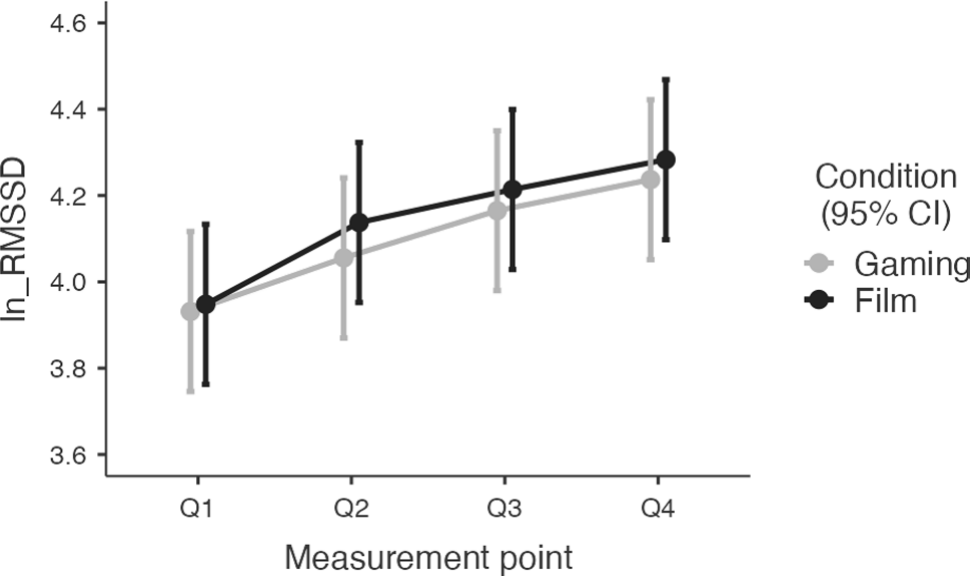
RMSSD responses to four measurement points. Q1 was quarter one, Q2 was quarter two, Q3 was quarter three, Q4 was quarter four of the sleep time

**Fig. 5:**
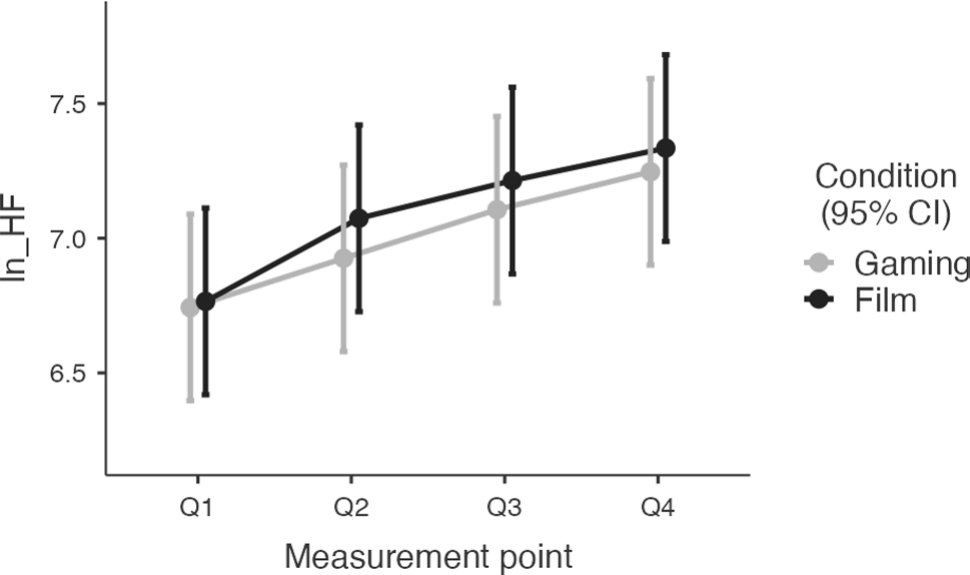
HF-HRV responses to four measurement points. Q1 was quarter one, Q2 was quarter two, Q3 was quarter three, Q4 was quarter four of the sleep time

For the statistical analysis, each heart rate, RMSSD and HF-HRV value was log-transformed using the natural logarithm (In + 1) to approximate normal distributions (Figs. 3, 4, 5). Normality tests confirmed the effectiveness of this transformation (see Supplemental Table S1).

First, baseline values of heart rate, HRV-RMSSD and HF-HRV in the gaming and film conditions were compared using a dependent *t*-test. Baseline values were defined as the data points recorded five minutes before the onset of Q1.

Second, the effect of the two conditions (gaming and film) on heart rate and HRV parameters (RMSSD and HF-HRV) was analyzed using a mixed model, with the interaction of measurement point Q1, Q2, Q3, and Q4 as a fixed effect. The intercept was treated as a random effect to account for intra-individual changes. The purpose of this analysis was to investigate whether the impact of condition on heart rate and HRV parameters varied across the four measurement time points.

Du to the absence of objective sleep stating using polysomnography (PSG) or actigraphy, the entire sleep period was divided into four equal segments (Q1–Q4). The measurement points refer to evenly divided segments of the total sleep duration, with each quarter representing 25% of an individual’s total sleep time. This segmentation allows for the analysis of changes in heart rate and HRV parameters over time. It should be noted that these quarters may include different sleep stages, which provides the advantage of capturing autonomic patterns across the entire sleep period.

To test whether the testing day (Day 1 vs. Day 2) affected heart rate and HRV parameters, testing day was included as a fixed effect in the model.

The software program used to analyze the data was *JAMOVI* (Gallucci, 2019).

## Results

### Heart Rate

There was no significant difference in baseline heart rate values between the gaming and film conditions (*t*(30) = 1.33, *p* = 0.192). The mixed model analysis revealed no significant effect of condition on heart rate (*t*(446.1) = − 1.22, *p* = 0.22; R^2^m = 0.117; R^2^c = 0.774; ICC = 0.744). There was no effect of testing day on heart rate (see Table 1). The interaction between condition and different measurement points (Q1–Q4) was not significant (see Table 1).

**Table 1:** Mixed model results of the fixed effect parameter of interaction between Heart Rate (HR) and Film-Gaming Condition, the time points Q1, Q2, Q3, Q4, the testing day, and between the conditions (film/gaming) and the measurement points (Q1–Q4)

Fixed effects parameter estimates								
Names	Effect	Estimate	95% Confidence interval		Uppe	df	t	*p*
			SE	Lower				
(Intercept)	(Intercept)	58.1613	1.343	55.529	60.794	30.0	43.3007	<.001
Measurement point1	Q2–Q1	− 5.3765	0.553	− 6.461	− 4.292	446.0	− 9.7157	<.001
Measurement point2	Q3–Q1	− 7.4354	0.553	− 8.520	− 6.351	446.0	− 13.4364	<.001
Measurement point3	Q4–Q1	− 7.5884	0.553	− 8.673	− 6.504	446.0	− 13.7128	<.001
Condition1	Film–Gaming	− 0.4766	0.392	− 1.245	0.291	446.1	− 1.2165	0.224
Testing Day1	2–1	0.0193	0.392	− 0.749	0.787	446.1	0.0492	0.961
Measurement point1 × Condition1	Q2–Q1 × Film– Gaming	0.5030	1.107	− 1.666	2.672	446.0	0.4545	0.650
Measurement point2 × Condition1	Q3–Q1 × Film–Gaming	0.3310	1.107	− 1.838	2.500	446.0	0.2991	0.765
Measurement point3 × Condition1	Q4–Q1 × Film–Gaming	0.1986	1.107	− 1.971	2.368	446.0	0.1795	0.858

### RMSSD

There was no significant difference in baseline RMSSD values between the gaming and film conditions (*t*(30) = − 0.27, *p* = 0.790). The mixed model analysis revealed a significant effect of condition on RMSSD with higher values in the film condition than in the gaming condition (*t*(446.1) = 2.05, *p* = 0.04; R^2^m = 0.051; R^2^c = 0.778; ICC = 0.766). There was no effect of testing day on RMSSD (see Table 2). The interaction between condition and different measurement points (Q1–Q4) was not significant (see Table 2).

**Table 2:** Mixed model results of the fixed effect parameter of interaction between RMSSD and Film-Gaming Condition, the time points Q1, Q2, Q3, Q4, the testing day, and between the conditions (film/gaming) and the measurement points (Q1–Q4)

Fixed effects parameter estimates								
Names	Effect	Estimate	SE	95% Confidence interval		df	t	*p*
				Lower	Upper			
(Intercept)	(Intercept)	4.12135	0.0860	3.95275	4.2899	30.0	47.9104	<.001
Measurement point1	Q2–Q1	0.15688	0.0334	0.09133	0.2224	446.0	4.6905	<.001
Measurement point2	Q3–Q1	0.24977	0.0334	0.18422	0.3153	446.0	7.4678	<.001
Measurement point3	Q4–Q1	0.32029	0.0334	0.25474	0.3858	446.0	9.5761	<.001
Condition1	Film–Gaming	0.04851	0.0237	0.00209	0.0949	446.1	2.0482	0.041
Testing Day1	2–1	0.01061	0.0237	− 0.03581	0.0570	446.1	0.4479	0.654
Measurement point1 × Condition1	Q2–Q1 × Film– Gaming	0.06538	0.0669	− 0.06573	0.1965	446.0	0.9773	0.329
Measurement point2 × Condition1	Q3–Q1 × Film– Gaming	0.03245	0.0669	− 0.09866	0.1636	446.0	0.4851	0.628
Measurement point3 × Condition1	Q4–Q1 × Film–Gaming	0.02961	0.0669	− 0.10150	0.1607	446.0	0.4427	0.658

### HF-HRV

There was no significant difference in baseline HF-HRV values between the gaming and film conditions (*t*(30) = − 0.12,* p* = 0.903). The mixed model analysis revealed a significant effect of condition on HF-HRV with higher values in the film condition than in the gaming condition (*t*(446.1) = 2.00, *p* = 0.05; R^2^m = 0.042; R^2^c = 0.762; ICC = 0.751). There was no effect of testing day on HF-HRV (see Table 3). The interaction between condition and different measurement points (Q1–Q4) was not significant (see Table 3).

**Table 3:** Mixed model results of the fixed effect parameter of interaction between HF-HRV and Film-Gaming Condition, the time points Q1, Q2, Q3, Q4, the testing day, and between the conditions (film/gaming) and the measurement points (Q1–Q4)

Fixed effects parameter estimates								
Names	Effect	Estimate	SE	95% Confidence interval		df	t	*p*
				Lower	Upper			
(Intercept)	(Intercept)	7.0512	0.1602	6.73723	7.365	30.0	44.01875	<.001
Measurement point1	Q2–Q1	0.2456	0.0647	0.11867	0.372	446.0	3.79311	<.001
Measurement point2	Q3–Q1	0.4060	0.0647	0.27912	0.533	446.0	6.27169	<.001
Measurement point3	Q4–Q1	0.5365	0.0647	0.40961	0.663	446.0	8.28732	<.001
Condition1	Film–Gaming	0.0918	0.0458	0.00197	0.182	446.1	2.00296	0.046
Testing Day1	2–1	0.0271	0.0458	− 0.06270	0.117	446.1	0.59216	0.554
Measurement point1 × Condition1	Q2–Q1 × Film–Gaming	0.1259	0.1295	− 0.12782	0.380	446.0	0.97272	0.331
Measurement point2 × Condition1	Q3–Q1 × Film–Gaming	0.0856	0.1295	− 0.16820	0.339	446.0	0.66083	0.509
Measurement point3 × Condition1	Q4–Q1 × Film–Gaming	0.0656	0.1295	− 0.18820	0.319	446.0	0.50636	0.613

## Discussion

Evening gaming with highly arousing video games was associated with a smaller increase in vagally mediated heart rate variability (vmHRV), including RMSSD and HF-HRV, during sleep compared to watching a nature film. Although vmHRV increased in both conditions, reflecting parasympathetic recovery, the film condition resulted in a significantly greater increase. The observed differences in vmHRV between conditions were statistically significant; however, the marginal R^2^ values (RMSSD: 0.051, HF-HRV: 0.042) suggest that the effect size is moderate. While both activities support autonomic recovery during sleep, the film condition may promote a more pronounced parasympathetic activation. This aligns with previous research indicating that high-intensity or emotionally engaging activities before bedtime can impair autonomic recovery (Chaput et al., 2011; Porter & Goolkasian, 2019). However, no significant differences in HR were observed between the gaming and film conditions, highlighting the importance of HRV as a more sensitive measure of subtle changes in autonomic regulation (Quigley et al., 2024).

The observed differences in vagally mediated HRV (vmHRV) between gaming and film conditions align with the Neurovisceral Integration Model (Thayer et al. 2009), which describes how prefrontal-autonomic interactions regulate self-regulation and recovery. The prolonged cognitive and emotional demands of gaming likely extended sympathetic activation, delaying parasympathetic recovery during sleep. While the Vagal Tank Theory (Laborde et al., 2018) offers a conceptual metaphor to describe resource depletion and replenishment, the Neurovisceral Integration Model provides a more physiologically grounded explanation for the observed autonomic modulation. Future research should further investigate how different types of pre-sleep cognitive load impact vagal recovery and subsequent sleep quality.

Contrary to the initial hypothesis, heart rate did not significantly differ in the night after the gaming and film-watching conditions. This aligns with findings from Weaver et al. (2010), who reported that physiological arousal remained unchanged during the condition when comparing thirteen male adolescents in experimental (active video gaming) and control (passive DVD watching) conditions (Weaver et al., 2010). However, the results differ from Higuchi et al. (2005), who observed significantly higher heart rates while playing video games compared to a control condition with a simple task with low mental load even 1.45 h later (Higuchi et al., 2005). These discrepancies may be explained by differences in study design, such as the gaming duration, intensity, and participant characteristics. In this study, HR changes may have been masked by the habituation of experienced gamers, highlighting the need for sensitive HRV metrics to detect autonomic shifts, as HR alone may not adequately capture the nuanced effects of gaming-induced arousal.

The persistent activation of the sympathetic nervous system during gaming may have moderated the increase in vmHRV compared to the film condition. Video games, particularly competitive or immersive ones, are known to elicit heightened arousal and stress responses (Long et al., 2023). Such sympathetic activation increases energy expenditure from the vagal tank, impairing the body’s ability to transition into a restorative parasympathetic state during sleep. By contrast, the nature documentary condition likely facilitated relaxation, supporting parasympathetic recovery and higher vmHRV during sleep. These findings underscore the relevance of pre-sleep activity selection in influencing autonomic regulation.

The inclusion of habitual gamers may have moderated the observed effects. As reviews and experimental studies indicate (De Rosa et al., 2024; Ivarsson et al., 2013), habitual gamers often exhibit attenuated physiological responses to gaming compared to casual or non-gamers, likely due to adaptation. This could explain why the increase in vmHRV during sleep, while smaller in the gaming condition, was not further diminished. Additionally, the exclusive use of highly arousing games (League of Legends and Counter Strike: Global Offensive) likely contributed to the observed larger difference in parasympathetic activity during sleep compared to the film condition. Highly arousing video games played before bedtime can delay sleep onset and alter sleep structure by increasing sympathetic nervous system activity (De Rosa et al., 2024). In contrast, non-arousing, cognitively challenging games have been shown to improve sleep continuity, stability, and organization. These effects depend not only on the level of arousal associated with the game but also on the duration and frequency of gaming sessions (De Rosa et al., 2024).

This study employed a highly standardized protocol, minimizing confounding variables such as daily form, mood, and environmental conditions. All participants performed both the gaming and control conditions on two consecutive days to minimize random effects, such as daily form fluctuations. Habitual gamers were selected to standardize gaming expertise and immersion levels, and strict inclusion criteria (e.g., no sleep disorders, cardiovascular-altering medications, or substance use) enhanced the reliability of the findings. These methodological strengths align with the recommendations of Welsh et al. (2023), who emphasized the importance of robust and standardized approaches in HRV research (Welsh et al., 2023).

The absence of an active control condition, such as a non-arousing video game or an arousing movie, represents a limitation in distinguishing between the effects of gaming and the calming influence of the documentary. While the documentary likely enhanced parasympathetic activity due to its relaxing properties, it is unclear whether the observed differences were driven primarily by the arousing nature of gaming or the strong calming effects of the documentary. The significantly stronger increase in parasympathetic activity during the control condition compared to the gaming condition underscores the need for future studies to include active control conditions to better isolate the effects of arousal levels and cognitive demands within the framework of the Vagal Tank Theory.

However, several limitations of this study should be noted. Despite efforts to standardize the gaming experience by initiating participants at their individual score levels, comparing the respective game challenges across participants remains a complex endeavor. This approach may not eliminate the dynamic nature of skill, focus, and adaptability that affects gameplay. Furthermore, the attempt to standardize subjects' daily activities encountered inherent limitations, potentially contributing to variations in the recorded cardiovascular parameters. The sample was composed solely of habitual gamers, who may exhibit attenuated physiological responses to gaming-induced stress due to habituation effects. Habitual gamers have been reported to show reduced autonomic reactivity compared to casual or non-gamers (Welsh et al., 2023), which might partly explain the absence of significant differences in heart rate between conditions. Future research should compare HRV responses between casual and habitual gamers to elucidate the role of gaming frequency and experience on autonomic recovery.

A major methodological drawback is the lack of objective sleep staging using PSG or actigraphy. HRV measures vary substantially with sleep stage; for example, non-REM sleep is typically characterized by increased parasympathetic activity, whereas REM sleep shows heightened sympathetic activity (Stein & Pu, 2012). Consequently, differences in the distribution of sleep stages across conditions could have contributed to the observed variations in vmHRV. Future studies should incorporate objective sleep staging methods to disentangle the effects of sleep architecture on autonomic recovery and better interpret HRV changes following different pre-sleep activities.

The study exclusively recruited male habitual gamers to control for the effects of the menstrual cycle and the use of oral contraceptive agents on sleep and HRV (Weaver et al., 2010). While this approach minimizes hormonal variability, it may also result in attenuated autonomic responses compared to a more diverse sample. Future studies should include female participants—using appropriate cycle tracking—to account for hormonal fluctuations.

Another methodological limitation is the lack of direct supervision during interventions, as adherence relied on self-reported documentation. Although participants logged session times, accuracy could not be objectively verified. Future studies should implement automated monitoring systems for improved compliance tracking. Additionally, the lack of self-report measures on arousal, pleasure, and engagement limits subjective assessment, and their inclusion is recommended to better evaluate individual experiences in both conditions.

This study underscores the importance of pre-sleep activity selection, particularly in habitual gamers, to support autonomic recovery and promote optimal sleep health. While the observed differences in vmHRV were statistically significant, the moderate effect size suggests that individual variability plays an important role in autonomic regulation. Future research should expand on these findings by investigating diverse gaming genres, their intensity levels, and their long-term effects on sleep and recovery.

## Supplementary Information

Below is the link to the electronic supplementary material.

Supplementary file1 (DOCX 93 kb)

## Data Availability

The data that support the findings of this study are not openly available due to reasons of sensitivity and are available from the corresponding author upon reasonable request.
