# Supplementary figures and images for: The Effects of Excessive Arousing Video Gaming on vmHRV During Sleep in Habitual Gamers
Source: Appl Psychophysiol Biofeedback. 2025 Jul 5;51(1):169–78. doi: 10.1007/s10484-025-09723-z (PMC12920350; doi:10.1007/s10484-025-09723-z)

**Supplemental Table 1**

**
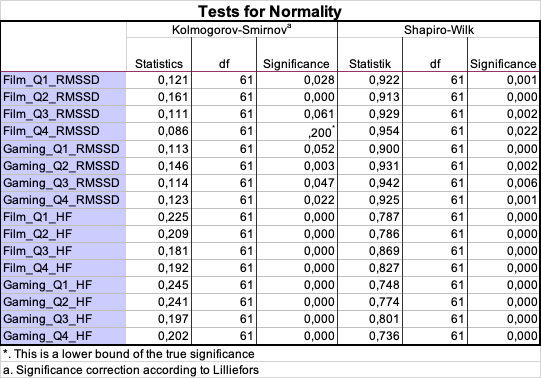
**

**Tab. 1** Normality before Logarithmic

**
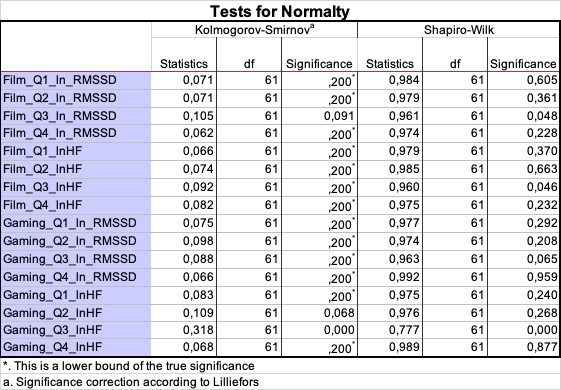
**

**Tab. 2** Normality after Logarithmic

Supplement: Supplementary file 1 — Supplementary file1 (DOCX 93 kb) [file 10484_2025_9723_MOESM1_ESM.docx]
